# Overview of Electromagnetic Interference Mechanisms and System-Level Effects in MHz-Range Wireless Charging for Electric Vehicle Applications

**DOI:** 10.3390/s26123891

**Published:** 2026-06-18

**Authors:** Kirill Nefjodov, Mahmoud Ibrahim, Anton Rassõlkin

**Affiliations:** Department of Electrical Power Engineering and Mechatronics, Tallinn University of Technology, 19086 Tallinn, Estonia; kinefj@taltech.ee (K.N.); mahmoud.mohamed@taltech.ee (M.I.)

**Keywords:** inductive power transmission, electric vehicles, electromagnetic interference, electromagnetic compatibility, power semiconductor devices, wireless charging

## Abstract

**Highlights:**

**What are the main findings?**

- MHz-range WPT for EVs introduces qualitatively new EMI mechanisms—fast SiC/GaN switching transitions, high-Q resonant current and voltage amplification, frequency-dependent parasitic capacitive coupling, and misalignment-driven magnetic-field redistribution—that are largely absent or negligible in conventional kHz-range systems.
- The lumped-element circuit assumptions used in most existing WPT analyses break down at MHz frequencies, where distributed electromagnetic behavior and structural coupling paths dominate the actual interference picture.

**What are the implications of the main findings?**

- Reliable MHz-range wireless EV charging requires frequency-aware electromagnetic design and integrated coupler-converter-vehicle modeling rather than the converter-centric EMI mitigation strategies developed for lower-frequency systems.
- Existing EMC standards and assessment methods, calibrated to kHz-range WPT, leave material gaps at MHz frequencies—both in test methodology and in the protection of nearby vehicle electronics, sensors, and ADAS subsystems—and need to be revisited for safe deployment.

**Abstract:**

Wireless power transfer (WPT) systems for electric vehicles (EVs) are increasingly being studied in the MHz range to increase power density and reduce the size of passive components. However, operation at higher frequencies significantly changes electromagnetic interference (EMI) behaavior. Fast switching in SiC- and GaN-based inverters, high-Q resonant operation, and frequency-dependent parasitic capacitances create conductive, capacitive, and magnetic interference mechanisms that are less significant in conventional kHz-range systems. Although many existing studies focus on power-transfer efficiency and converter optimization, EMI mechanisms in MHz-range EV WPT systems remain insufficiently systematized from a system-level electromagnetic perspective. This paper presents a state-of-the-art review of EMI generation mechanisms and system-level effects in high-frequency WPT systems for electric vehicles. The review considers the main interference sources and coupling paths, including switching-induced common-mode currents, resonant amplification of current and voltage stress, capacitive coupling between the coupler and nearby conductive structures, and magnetic-field redistribution caused by coil misalignment. Special attention is given to the transition from lumped-element assumptions to more distributed electromagnetic behavior at higher frequencies. The review also discusses the possible impact of these mechanisms on vehicle electronic subsystems and highlights the need for frequency-aware electromagnetic design, integrated modeling, and more rigorous EMC assessment for reliable MHz-range wireless EV charging systems.

## 1. Introduction

The electrification of road transport has accelerated in recent years. According to the Global EV Outlook 2025, electric car sales exceeded 20 million worldwide in 2025, accounting for more than 22% of new car sales, while the global electric car fleet reached almost 60 million by the end of the year [1]. This rapid growth places charging infrastructure at the center of transport electrification, and among the available approaches, wireless charging is attracting increasing attention as a convenient and contactless alternative to conventional plug-in systems.

Despite this rapid deployment, conductive (plug-in) charging continues to exhibit practical limitations that motivate the search for contactless alternatives. Reliance on physical connectors introduces mechanical wear, exposure to moisture and dust, and safety considerations during connection and disconnection, while the need for user interaction limits compatibility with autonomous and shared-mobility platforms [2]. High-power cables also become heavy and difficult to handle as charging power increases, and outdoor connectors in cold or wet climates raise additional reliability and safety concerns. Wireless power transfer (WPT) addresses these limitations by enabling energy transfer across an air gap without a galvanic connection, supporting both stationary charging in parking environments and, in principle, dynamic charging during vehicle motion [3].

WPT systems for EV applications are mainly based on near-field power transfer methods, particularly inductive power transfer and magnetic resonant coupling. These approaches enable non-radiative energy transfer across short air gaps and can achieve high efficiency under proper coupling and alignment conditions [4,5].

In practical automotive implementations, inductive wireless charging systems typically operate in the 20–200 kHz range, while resonant inductive configurations commonly operate in the 85–150 kHz band [4]. Within this frequency domain, SAE J2954 defines the primary operating framework for the 85 kHz band in stationary wireless EV charging systems and addresses key aspects such as interoperability, safety, and system performance [6], with associated EMC compliance requirements for inductive automotive charging analyzed in detail by Sulejmani et al. [6]. Complementary international frameworks include IEC 61980-1 [7], which specifies general requirements and EMC provisions for EV WPT supply devices up to 1000 V AC and 1500 V DC [8], and CISPR 25, which establishes limits and measurement procedures for on-board radio disturbances over the 150 kHz–2.5 GHz range applicable to all vehicle-installed electronic components [9]. ANSI C63.30 [9] additionally provides WPT-specific test methods that address measurement scenarios not fully covered by general automotive EMC standards [10]. Although stationary inductive charging has reached a higher level of standardization, technical and regulatory frameworks for dynamic and higher-power wireless charging remain less mature [4]. Comprehensive recent reviews of EV WPT technology, including the surveys by Mahesh et al. of inductive WPT charging architectures [11], Ahmad et al. of stationary and dynamic charging strategies [12], and Sagar et al. of recent developments in wireless power transfer technologies for EV charging systems [13], have consistently identified electromagnetic interference and electromagnetic compatibility as central design constraints, while the state-of-the-art survey of automotive inductive power transfer by Cirimele et al. positions MHz-range operation as a key direction for next-generation systems [14]. Early foundational treatments of WPT for EVs, including the technology overview by Vilathgamuwa and Sampath covering static and dynamic charging principles [15], the survey by Korakianitis et al. of IPT and magnetic-resonance-based charging topologies and compensation strategies [16], and the multi-technology review by Triviño et al. covering inductive, capacitive, radiofrequency, and laser-based approaches applied to EVs [17], together provide the broad technological context within which the EMI challenges addressed in the present paper arise. Building on this background, recent research has begun to explore wireless power transfer at MHz frequencies, extending beyond the conventional sub-100 kHz range used in most EV charging systems. For example, Wu et al. demonstrated a 6.78 MHz resonant WPT system based on a GaN Class EF inverter, reporting a peak DC-DC efficiency of 84.6% with compact coil structures [18]. Although this prototype was not developed specifically for EV charging, it demonstrates the technical feasibility of resonant MHz-range WPT operation. Wide-bandgap semiconductor devices such as silicon carbide (SiC) and gallium nitride (GaN) further support this transition by enabling higher switching frequencies with lower switching losses than conventional silicon devices, while also reducing the size of passive components and heat sinks [19]. Several MHz-range systems, tested for, or directly relevant to, EV charging, illustrate the practical feasibility of this transition across a range of coupling methods, frequencies, and power levels. Qin et al. reported an inductive system operating at 3 MHz that transferred 2.4 kW across a 100 mm air gap at 93.2% DC-DC efficiency using a multi-layer non-uniform self-resonant coil and a GaN power stage [20]. Phan et al. demonstrated a 6.78 MHz, 1 kW inductive link with air-core coils intended for electric transportation, eliminating the heavy litz wire and ferrites typical of low-frequency designs [21]. Beyond inductive coupling, Regensburger et al. demonstrated a large-air-gap capacitive WPT system for EV charging at 13.56 MHz [22], while Gu and Rivas-Davila achieved 1.7 kW at 6.78 MHz with low-cost air-core coils, maintaining DC-DC efficiency above 93% from 0.1 to 1.7 kW, peaking at 95.7% [23]. Earlier work by Liu, Fu and Ma established analytical design procedures for 6.78 MHz Class-E current-driven rectifiers that have informed subsequent multi-MHz inverter–rectifier co-design [24], and Arteaga et al. demonstrated dynamic load tracking in multi-MHz inductive power transfer systems operating at 6.78 MHz, illustrating that MHz-range coupling remains stable under significant load and coupling variation [25]. At the system level, Sasatani et al. reported a room-scale magnetoquasistatic wireless power transfer cavity that delivers multi-watt power over volumes large enough to encompass a vehicle, indicating possible architectures for distributed charging environments [26]. Together with the 6.78 MHz GaN Class-EF prototype of Wu et al. [18], these systems span inductive and capacitive coupling from 3 to 13.56 MHz at power levels up to several kilowatts, confirming that MHz-range WPT is technically viable for EV-relevant power and gap conditions.

Operation at higher frequencies moves wireless power transfer beyond a purely quasi-static magnetic description and increases the importance of parasitic and frequency-dependent electromagnetic effects [27]. In EV wireless charging systems, the voltage distribution across the coil turns can also contribute to electric-field formation in addition to the intended magnetic coupling. Recent coil design studies show that conventional turn arrangements are associated with higher E-field emissions, whereas phase-opposed voltage patterns between adjacent turns can reduce local electric-field intensity, highlighting the role of coil voltage distribution in EMI and EMF performance [28]. High-frequency switching in EV power electronic converters is a well-established source of EMI, contributing to both conducted and radiated disturbances in electrified vehicle systems [29]. In wireless charging systems, parasitic capacitances and nearby conductive structures can create unintended common-mode coupling paths, and these effects become more influential as frequency increases [27,28]. In addition, multicoil and shielding configurations can significantly modify the near-field distributions around the vehicle, indicating that field behavior and emission patterns should be evaluated at the system level rather than at the level of an isolated coil or converter [30].

Existing studies suggest that operating in the MHz range can increase the electromagnetic complexity of WPT systems due to stronger parasitic effects, higher switching frequencies, and more stringent EMC requirements [28]. At the same time, the available literature remains more developed in the areas of efficiency optimization, compensation design, and converter implementation than in the systematic analysis of EMI generation and system-level electromagnetic interactions [31].

As a result, EMI research in EV wireless charging still appears fragmented, and a unified mechanism-based understanding of EMI generation and its system-level effects in MHz-range WPT architecture has not yet been clearly established [32].

This paper presents a state-of-the-art review of electromagnetic interference (EMI) mechanisms and system-level electromagnetic effects in MHz-range wireless power transfer systems for electric vehicles. Unlike efficiency-driven surveys, this work adopts a mechanism-oriented electromagnetic perspective and focuses on how the transition from quasi-static to more distributed high-frequency behavior affects coupling paths, vehicle-level interactions, and EMC performance.

The primary objectives of this review are:

- To identify and classify dominant EMI generation mechanisms in MHz-range WPT systems;
- To analyze frequency-dependent coupling pathways, distributed parasitic effects, and common-mode propagation phenomena;
- To establish a system-level interaction framework linking coil-level electromagnetic behavior to EV electronic architecture;
- To evaluate the impact of MHz-related electromagnetic effects on vehicle subsystems and surrounding electronics;
- To highlight design implications and open research challenges for high-frequency WPT integration.

## 2. Fundamentals of Wireless Power Transfer (WPT) in MHz Range

**Figure 1:**
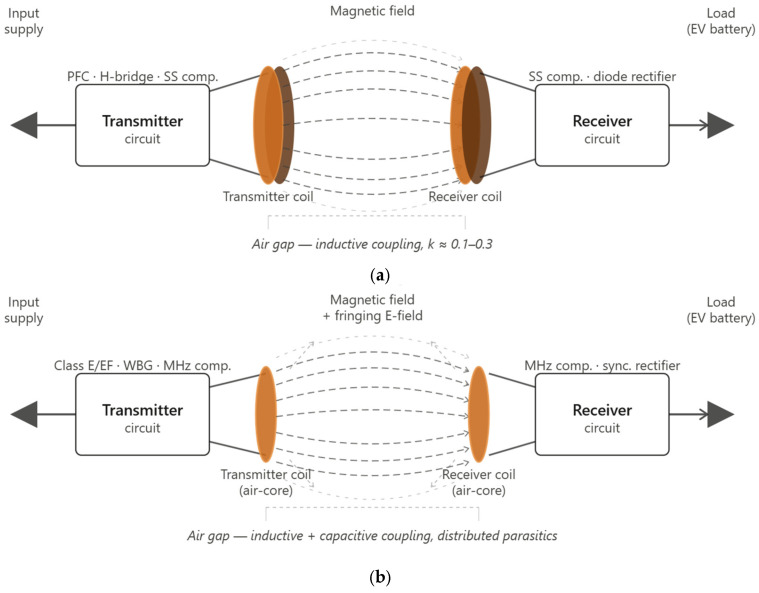
Conceptual contrast between (**a**) conventional kHz-range and (**b**) emerging MHz-range EV wireless power transfer, highlighting the shift in coil arrangement and electromagnetic field behavior.

In weakly coupled resonant WPT systems, analytical results show that the maximum transferable power in an idealized configuration scales with the square of the angular frequency, which helps explain the interest in higher-frequency operation. At the same time, Andersen et al. showed that parasitic coil capacitance $C_{p}$ can significantly affect receiver dynamics and output power, and that the optimal operating conditions depend on the intrinsic electrical parameters of the coil, including  L, R, and $C_{p}$ [30]. As the operating frequency increases toward the MHz range, these parasitic effects become more influential and reduce the validity of simplified lumped-element assumptions. Experimental results reported by Woo et al. further show that tuning the receiver resonance changes the phase relationship between transmitter and receiver currents and, under the reported operating conditions, reduces the leakage magnetic field by 32.41% [31]. This indicates that magnetic field distribution in resonant WPT systems depends not only on geometry, but also on tuning conditions and current phase. Taken together, these findings show that MHz-range operation creates stronger coupling between frequency, parasitic effects, resonant tuning, and spatial field behavior. Under such conditions, the electromagnetic response of the system can no longer be described adequately within a purely quasi-static magnetic framework. Figure 1 highlights the difference between conventional kHz-range EV WPT and emerging MHz-range architectures.

Table 1 summarizes the principal differences between conventional kHz-range and emerging MHz-range WPT operation that motivate this shift from a modeling perspective.

### 2.1. Inductive Power Transfer Principle and Validity Limits

**Figure 2:**
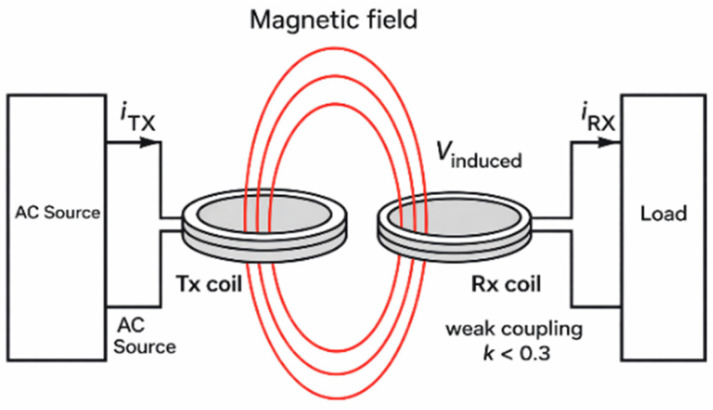
Equivalent representation of an inductive power transfer (IPT) system.

In resonant inductive WPT systems, a high-frequency inverter drives an alternating current in the transmitter (TX) coil, producing a magnetic field that links the receiver (RX) coil across an air gap [33]. The resulting time-varying magnetic flux Φ(t) induces a voltage in the RX coil according to Faraday’s law:

(1) $$v_{r}=-N_{r}\frac{d\Phi \left(t\right)}{dt},$$

where $N_{r}$ is the number of turns in the receiving coil and Φ(t) is the magnetic flux linking the transmitter and receiver coils [33]. The induced voltage drives a current in the secondary circuit, enabling contactless transfer of electrical energy from the source to the load. A simplified representation of an inductive WPT system is shown in Figure 2. The transmitting and receiving coils are connected via compensation networks that counteract the coils’ inductive reactance under high-frequency excitation, thereby enabling resonant operation and effective power transfer across the air gap [33].

In practical EV wireless charging systems, the transmitting and receiving coils are separated by a substantial vertical air gap determined by vehicle ground clearance and alignment tolerance. This separation reduces magnetic flux linkage and limits the achievable magnetic coupling between the coils. In addition to standardized automotive clearances, commercial implementations have also reported air-gap tolerances of up to approximately 12 inches (about 300 mm) in specific wireless charging systems [34].

The magnetic interaction is quantified by the mutual inductance M, which is commonly expressed as:

(2) $$M=k\sqrt{L_{T}L_{R}},$$

where $L_{T}$ and $L_{R}$ denote the self-inductances of the transmitting and receiving coils, and k is the coupling coefficient, with 0≤k≤1 [35]. For EV wireless charging geometries involving substantial air gaps and practical alignment tolerances, the coupling coefficient typically remains well below unity, reflecting weak magnetic interaction and increased sensitivity to displacement. Zhang et al. [36,37,38] reported that mutual inductance decreases under spatial misalignment, with the exact reduction depending on coil topology, excitation mode, and displacement direction. In their analysis of polarized EV couplers, the decrease was on the order of 25–30% for *x*-axis displacement and about 56–70% for *z*-axis displacement, while the *y*-axis response followed a different trend [36]. These results indicate that practical EV wireless charging systems operate under weak and displacement-sensitive magnetic coupling conditions. Under a series–series compensated resonant model with lumped parameters and resistive loading, the transferred load power depends on both the operating angular frequency and the mutual inductance. As shown by Zakerian et al. [32], the analytical expression for transferred load power contains a numerator proportional to $\omega^{2}M^{2}$. Under idealized resonant conditions, this leads to a dominant quadratic trend that can be expressed in simplified form as:

(3) $$P_{\mathrm{out}}\propto \omega^{2}M^{2},$$

indicating that higher operating frequency and stronger magnetic coupling increase the theoretical power transfer capability. However, because practical EV wireless charging systems operate with limited coupling, inductive interaction alone is generally insufficient to maintain effective power transfer with high efficiency. Resonant compensation is therefore required to sustain effective operation under weak-coupling conditions. Conventional IPT analysis is usually based on simplified assumptions such as dominant magnetic near-field interaction, lumped-element coil modeling, and negligible capacitive effects. These assumptions remain useful for conventional kHz-range EV charging systems, where magnetic coupling dominates the transfer process. As the operating frequency increases, however, parasitic capacitive and other frequency-dependent electromagnetic effects become more important, reducing the accuracy of simplified magnetic models for full system-level descriptions [37]. In addition, analytical studies of EV wireless charging couplers often rely on magnetoquasistatic formulations under specific operating conditions, showing that the applicability of classical IPT assumptions depends on both the frequency range and the modeling scope [36]. Table 2 summarizes the main limitations of these assumptions at higher frequencies.

From the above Table 2, notable, stray capacitance and stray inductance—negligible in kHz-range systems where the coil dimensions are a small fraction of the operating wavelength—become significant at MHz frequencies, where inter-turn and coil-to-chassis stray capacitances introduce unintended common-mode coupling paths and stray inductance in interconnects produces distributed resonances. These effects directly influence field distribution, coupling behavior, and unintended electromagnetic interactions, thereby establishing the physical basis for EMI mechanisms in high-frequency WPT systems.

### 2.2. Resonant Compensation Under MHz Constraints

In weakly coupled EV wireless power transfer systems, resonant compensation networks are used to maintain effective power transfer across substantial air gaps. By compensating the coil reactance, these networks bring the system close to resonance and improve power transfer capability. At higher operating frequencies, however, resonant behavior becomes more sensitive to parasitic effects and frequency-dependent losses, which makes tuning more difficult than in conventional kHz-range systems [37]. At MHz frequencies, distributed and parasitic effects become increasingly important, so small variations in effective inductance or capacitance can produce noticeable detuning. In addition, parasitic capacitances associated with coils, shielding structures, and nearby conductors can aggravate conductive common-mode behavior and complicate EMC compliance [28,37]. The nominal resonant frequency of the compensation network is defined by:

(4) $$\omega_{0}=\frac{1}{\sqrt{LC}},$$

(5) $$f_{0}=\frac{1}{2\pi \sqrt{\mathrm{LC}}},$$

where L and C denote the nominal inductance and capacitance of the resonant network. In practical MHz-range EV WPT systems, the effective values of these parameters deviate from their nominal values because of distributed and parasitic contributions, making resonant tuning inherently more sensitive than in lower-frequency systems [37]. While resonant operation is essential in inductive WPT systems, the selected compensation topology affects the equivalent input impedance and current distribution under varying load and coupling conditions [38]. Common EV-oriented compensation configurations include series–series (SS), LCL, and LCC networks [39]. Comparative analysis shows that the SS topology is suitable for high-power operation but may experience a significant increase in primary-side current when mutual inductance is reduced [39]. In contrast, the LCL topology provides more robust and stable operation, although it transfers less power [39]. The LCC topology retains good robustness while achieving significantly higher transferred power than LCL under comparable operating conditions [39]. These results indicate that the compensation topology is an important factor in determining current stress, robustness, and transmission performance under variations in coupling. Since these parameters define the electrical operating conditions of the resonant WPT system, topology selection is also relevant for subsequent electromagnetic analysis. However, the direct relationship between specific compensation topologies and electric-field emission patterns requires more targeted investigation. Table 3 summarizes the comparative current and power-transfer behavior of the main compensation topologies discussed for EV wireless charging systems. The reported results show that topology selection strongly affects robustness, current stress, and transmission performance under reduced mutual inductance and off-nominal operating conditions.

Beyond compensation topology, the performance of a resonant WPT system is fundamentally governed by the magnetic coupling coefficient k and the quality factors of the transmitting and receiving coils. The quality factor characterizes the ratio of stored energy to dissipated energy in a resonant circuit [40]. For a series-equivalent resonant circuit, the unloaded quality factor is expressed as:

(6) $$Q_{T}=\frac{\omega_{0}L_{T}}{R_{T}},$$

(7) $$Q_{R}=\frac{\omega_{0}L_{R}}{R_{R}},$$

where $\omega_{0}$ is the angular resonant frequency, $L_{T}$ and $L_{R}$ denote the self-inductances of the transmitter and receiver coils, and $R_{T}$ and $R_{R}$ represent their effective series resistances [40].

At MHz operating frequencies, the maximum achievable efficiency of a two-coil resonant WPT system is strongly dependent on the magnetic coupling coefficient k and the quality factors of the transmitter and receiver coils [41]. Under optimal loading conditions, the theoretical upper bound of transfer efficiency can be approximated as:

(8) $$\eta_{max}\approx \frac{k^{2}Q_{T}Q_{R}}{{\left(1+\sqrt{1+k^{2}Q_{T}Q_{R}}\right)}^{2}},$$

which indicates that efficiency scales with the product $k^{2}Q_{T}Q_{R}$ [41]. In the analyzed MHz IPT framework, the coil quality factor is defined as, directly linking transfer efficiency to the effective resistive loss of the resonant coils [41]. A reduction in coil quality factor therefore lowers the maximum achievable transfer efficiency, making coil Q key parameter governing wireless-link performance in MHz-range WPT systems.

### 2.3. WPT System Architecture and Frequency-Dependent EMC Behavior

An EV wireless power transfer system is typically implemented as a multi-stage power-electronic architecture that converts grid AC power into regulated DC power for battery charging. In representative configurations, the power path includes an AC/DC front-end with power-factor correction, a DC link, a high-frequency inverter, a resonant compensation network with transmitting and receiving coils, and a receiver-side rectification and regulation stage connected to the battery system [42].

**Figure 3:**
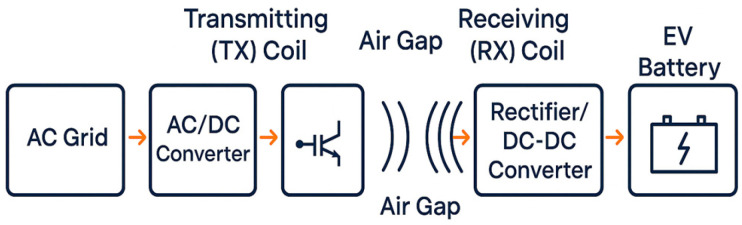
Simplified structure of a wireless power transfer (WPT) system for electric vehicles.

Figure 3 illustrates the simplified structure of such a system. The DC link supplies the high-frequency inverter, which excites the transmitting coil through the resonant network. Power is then transferred across the air gap by magnetic coupling and converted on the vehicle side into controlled DC power for battery charging [42].

From an EMC perspective, these stages should not be treated as fully independent functional blocks. In high-voltage electric drive systems, fast inverter switching generates large currents that excite parasitic elements in the power circuit and produce conducted disturbances propagating through both common-mode and differential-mode paths [43]. Because subsystem-only analysis yields limited predictive accuracy, the DC link, inverter, interconnections, and coupled power-transfer structure should be considered part of an integrated electromagnetic system [43].

In wireless charging systems, the coupler and its surrounding structures also contribute directly to EMC behavior. Conventional planar coil configurations can generate significant electric-field leakage, while shielding-based mitigation may introduce additional parasitic capacitance and aggravate conductive common-mode noise [28]. As a result, interference in MHz-range WPT systems propagates through multiple mechanisms simultaneously, including conductive paths through wired interconnections, capacitive coupling through parasitic capacitances, and inductive or leakage-field coupling associated with the transmitter–receiver structure [43]. These interactions motivate a system-level EMC perspective for high-frequency EV WPT systems. Table 4 and Table 5 summarize the main functional stages and EMI propagation mechanisms relevant to EV wireless charging systems. Taken together, the reviewed sources indicate that high-frequency WPT behavior cannot be assessed only at the level of an isolated inverter, coupler, or converter stage. Instead, the interaction between power electronics, resonant structures, interconnections, and surrounding conductive elements should be considered jointly, especially when evaluating EMC behavior in MHz-range EV WPT systems.

## 3. EMI Generation Mechanism in MHz-Range WPT Systems

In MHz-range WPT systems, EMI should be analyzed as a system-level phenomenon rather than as the result of an isolated component. A typical high-frequency WPT system includes a high-frequency inverter, a compensation network, and magnetically coupled transmitting and receiving coils [37]. Fast switching in the inverter generates large dv/dt and di/dt, excites parasitic elements in the power circuit, and produces conducted disturbances that propagate through electrical interconnections [43]. At the coupler level, conventional planar coil configurations can generate significant electric-field leakage, while shielding-based mitigation may introduce additional parasitic capacitance and aggravate conductive common-mode noise [28]. Therefore, EMI generation in MHz-range WPT systems should be evaluated by considering the interaction between the inverter, the resonant network, the coupling structure, and the associated parasitic propagation path [43].

### 3.1. High-Frequency Switching Transitions in SiC/GaN Inverters

Wide-bandgap (WBG) semiconductor devices such as GaN and SiC are important candidates for high-performance power conversion because their fundamental material properties differ substantially from those of conventional silicon [44]. Selected material parameters relevant to power-device performance are summarized in Table 6.

**Figure 4:**
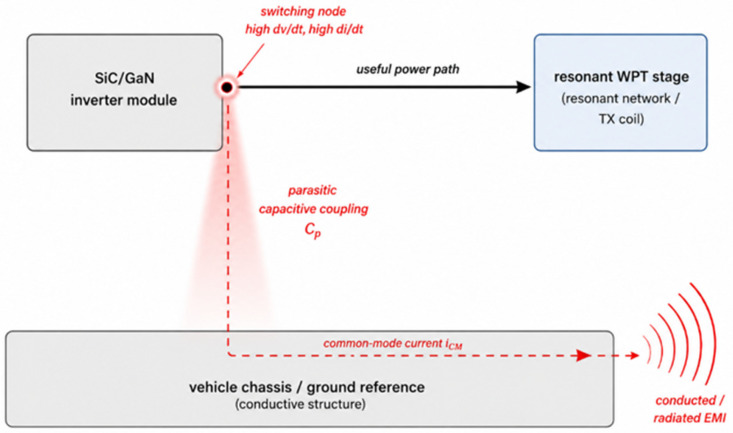
Simplified source–path representation of common-mode EMI excitation caused by fast switching in a SiC/GaN inverter stage of an MHz-range WPT system.

As shown in Table 6 above, GaN and SiC exhibit wider band gaps and much higher critical electric fields than silicon. The higher critical electric field allows the same breakdown voltage to be sustained with a substantially thinner drift region; for SiC and GaN, the drift region can be about ten times smaller than in silicon for the same breakdown voltage. These material characteristics are also associated with superior on-resistance and smaller device size compared with silicon-based power devices [44]. WBG devices enable faster switching at higher switching frequencies with lower switching losses and higher power density than conventional silicon-based devices [19]. Surakitbovorn and Rivas-Davila have shown that Class-E power amplifiers based on GaN HEMTs can be optimized for megahertz-range operation by accounting for nonlinear output capacitance and gate-charge effects that dominate device loss at MHz frequencies [45]. Kjærsgaard et al. further developed an analytical model of dynamic power losses in enhancement-mode AlGaN/GaN HEMTs operating in the 1–5 MHz range, identifying additional loss mechanisms that are not captured by conventional manufacturer data sheets [46]. More broadly, Wang et al. provide a comprehensive review of very-high-frequency (30–300 MHz) power converter technologies, magnetics, and resonant gate-drive strategies that are directly applicable to MHz-range WPT inverter stages [42,47]. This makes WBG devices attractive for high-frequency inverter stages, including those considered in MHz-range WPT systems. Rapid switching transitions in WBG-based converters are a major source of conducted electromagnetic interference. In GaN half-bridge converters, high dv/dt and di/dt are generated at the phase-leg switching node, and the drain-source voltage $V_{DS}$ and drain current $I_{D}$ are treated as the relevant common-mode noise sources in the behavioral model of. The corresponding common-mode current was experimentally modeled and validated over the 150 kHz–30 MHz frequency range [48]. In the context of MHz-range WPT systems, this mechanism can be represented as a source–path interaction: the WBG inverter switching node acts as the high-dv/dt) and high-di/dt noise source [49], while parasitic capacitance to the vehicle chassis provides a coupling path for common-mode current excitation, as illustrated in Figure 4.

High dv/dt operation increases the influence of parasitic capacitive couplings within the converter structure, thereby strengthening unintended common-mode and differential-mode current paths [50]. Taken together, these studies indicate that, in high-frequency WBG converters, EMI behavior is governed primarily by switching transitions and the parasitic coupling network rather than by the fundamental output frequency alone. As shown conceptually in Figure 4, high dv/dt switching excites parasitic capacitances between the switching node and chassis or ground, generating displacement currents that form a common-mode (CM) emission path. The resulting current through a parasitic capacitance $C_{p}$ is given by:

(9) $$i_{C}=C_{p}\frac{dV}{dt},$$

which directly links switching slew rate to CM current magnitude [50]. In GaN half-bridge converters, the phase-leg node is identified as the main location where fast voltage and current transients excite CM noise; the drain-source voltage $V_{DS}$ and drain current $I_{D}$ are treated as the relevant noise sources in the behavioral model of [48]. Complementary modeling approaches developed for WPT systems specifically—for example, the analytical frequency-domain transfer-function model of El-Shafie et al. for a Qi-compliant inductive WPT system—show that the conducted CM EMI spectrum can be predicted accurately from the time-domain noise-source voltage by combining a measured noise-source spectrum with an analytically derived coupling-path transfer function [51]. As illustrated in [50], increasing the DC-link voltage raises the overall magnitude of the noise-source spectrum; increasing the switching frequency shifts the lower spectral corner toward higher frequencies; and reducing the rise time shifts the upper corner frequency further upward. Accordingly, faster voltage transitions extend the spectral content into the MHz range and enhance the excitation of parasitic-capacitance-driven CM currents.

Overall, WBG devices enable fast switching transitions, and the resulting steep voltage edges broaden the high-frequency content of the switching waveform. In converter structures containing unavoidable parasitic capacitances, this leads to stronger CM current excitation and greater EMC sensitivity. For MHz-range WPT inverters, this implies that EMI performance is governed mainly by transient waveform characteristics and parasitic coupling paths rather than by nominal operating frequency alone. Table 7 highlights the influence of switching parameters on high-frequency noise excitation.

### 3.2. Magnetic Field Leakage Due to Coil Misalignment

**Figure 5:**
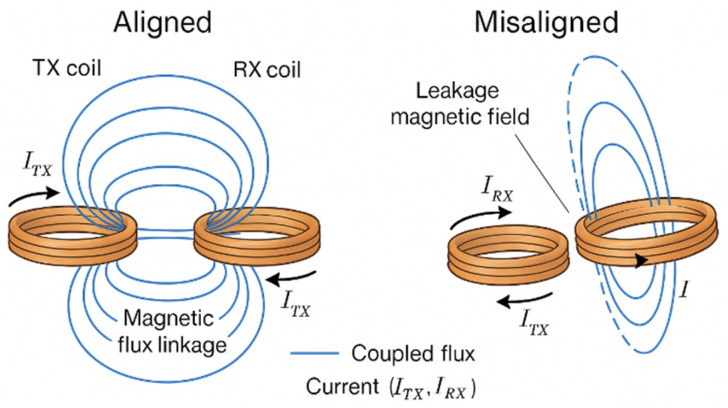
Magnetic flux distribution in WPT coils: ideal alignment, lateral misalignment showing magnetic field leakage.

In wireless power transfer systems, the transmitted power depends on the magnetic coupling between the transmitting and receiving coils. Under aligned conditions, magnetic flux is more effectively linked between the coils, which supports higher mutual inductance and more stable power transfer. When lateral, axial, or rotational misalignment occurs, the flux distribution becomes asymmetric, and a larger portion of the magnetic field extends outside the intended coupling region, increasing leakage magnetic-field components as illustrated in Figure 5.

Misalignment directly reduces mutual inductance and coupling coefficient because the flux linkage between the coils becomes incomplete. In an EV wireless charging study, Jeebklum et al. showed that lateral misalignment reduces magnetic flux density on the receiver coil, decreases mutual inductance and coupling coefficient, and lowers charging efficiency as the offset increases [47]. As additional quantitative support from another WPT application domain, Wen et al. reported that rotational misalignment produced a minimum coupling coefficient of k=0.266 and a maximum mutual-inductance fluctuation of 2.3%, while axial displacement of up to 40 mm caused a 6.3% mutual-inductance fluctuation [48,52,53].

These findings indicate that coil misalignment not only weakens effective coupling but also modifies the spatial distribution of the magnetic field, increasing stray components outside the intended transfer region. Table 8 illustrates the influence of misalignment on magnetic coupling.

Coil misalignment also modifies the spatial distribution of the magnetic field generated by the WPT system. Under non-ideal positioning, magnetic flux becomes asymmetric and extends beyond the intended coupling region, increasing stray magnetic-field components in the surrounding space. Finite-element analysis by Jeebklum et al. shows that lateral displacement alters the magnetic-flux-density distribution on the receiver coil, producing asymmetric field contours compared with the aligned condition [47]. Wen et al. further show that rotational and axial misalignment alter the magnetic-field pattern of the coupler, confirming that misalignment changes field distribution rather than only reducing coupling [48]. Therefore, misalignment should be interpreted not only as a reduction in coupling efficiency, but also as a field-redistribution mechanism that increases the relevance of leakage-field control in WPT systems.

The geometric mechanism by which misalignment redistributes magnetic flux is fundamentally independent of operating frequency: lateral, axial, or rotational displacement of the coils reduces flux linkage and causes a larger portion of the field to extend outside the intended coupling region regardless of whether the system operates at kHz or MHz. However, the electromagnetic consequences of this redistribution differ substantially between the two frequency regimes. In conventional kHz-range systems (20–150 kHz), the leakage field is quasi-static in character, and the primary concerns are biological exposure compliance with ICNIRP limits [54] and efficiency degradation due to reduced mutual inductance. The leakage field couples weakly into stray capacitances at these frequencies and does not readily excite common-mode conduction paths. At MHz frequencies, the same geometric redistribution produces leakage fields whose frequency is sufficiently high that they interact more strongly with parasitic capacitances between the coil structures, chassis, and nearby conductors. This interaction can drive common-mode currents through stray coupling paths that are absent or negligible in kHz-range operation. Additionally, the higher frequency means that the leakage field occupies a larger fraction of the operating wavelength, increasing the potential for near-field coupling to vehicle electronics. The shielding strategies effective at kHz—such as the SS-coil approach of Son et al. [50] operating under SAE J2954 conditions—rely on inductive cancelation tuned to a fixed frequency and may require re-evaluation when applied at MHz, where the broader spectral content and stronger parasitic coupling alter the effective impedance of the shielding path. In summary, misalignment-driven leakage is present at both kHz and MHz, but the MHz regime amplifies its electromagnetic consequences through stronger stray capacitive coupling and extended near-field interaction.

Under misaligned conditions, the magnetic field distribution between the TX and RX coils becomes asymmetric, and a larger portion of the magnetic flux extends outside the intended coupling region. This spatial redistribution increases stray magnetic-field components and makes leakage-field control more important in WPT systems [52,53]. In EV wireless charging, leakage magnetic field and misalignment are also relevant because they are associated with power-transfer efficiency reduction and potential malfunction of nearby electronic devices [55]. Leakage magnetic field generated under coil misalignment can be mitigated through auxiliary shielding structures. Son et al. proposed a shielding sensor (SS) coil integrated into an EV WPT system based on the SAE J2954 model [50]. When the SS coil operates in the inductive region, its current has an approximately 180° phase difference relative to the magnetic field generated by the WPT system, enabling partial magnetic-field cancelation [55]. In simulation, the proposed configuration achieved a shielding effect of up to 76% relative to the reference model, with a corresponding 3% reduction in power-transfer efficiency [55]. Experimental results at reduced power confirmed the same qualitative trend, although with a lower absolute shielding effect [55]. These results show that leakage-field mitigation under misalignment can be achieved, but not without trade-offs. Auxiliary shielding structures can reduce stray magnetic fields, while the achievable mitigation level depends on the additional current introduced into the auxiliary path and the corresponding efficiency [55].

### 3.3. High-Q Resonant Circulating Currents as EMI Amplifiers

In resonantly compensated high-frequency WPT systems, coil current is a key design variable because it directly determines the generated magnetic field [56]. At the same time, resonant operation is sensitive to tuning conditions: detuning in three-coil compensated systems affects output power, efficiency, and rectifier operating mode [57], while in LCC-S compensated WPT, the receiving-path current reaches its maximum under tuned conditions. Therefore, resonant compensation should not be viewed only as an efficiency-enabling mechanism. It also governs the magnitude of internal resonant currents and the associated electrical stress in coils and compensation elements. From an EMC perspective, this behavior is important because the current magnification along the resonant path strengthens the system’s magnetic field and increases sensitivity to perturbations in tuning and coupling conditions. As a result, resonant operation can intensify the physical conditions under which electromagnetic interference becomes more pronounced, especially when deviations from the intended operating point alter internal current distribution and stress levels [58]. As summarized in Table 9, current magnification in resonant WPT networks is not merely a circuit-level feature, but a practical operating condition governed by coil-current design, resonant tuning, and compensation state. Once internal current increases, the physical basis is created for stronger magnetic field excitation and higher current stress in the conductive path. In this sense, high-Q resonant operation should be treated as an important circuit-level precursor to EMC-related problems in high-frequency WPT systems.

The resonant current level is directly relevant to the electromagnetic behavior of high-frequency WPT systems. Li et al. showed that detuned compensation can attenuate the current in weakly coupled or unloaded transmitter coils, thereby reducing magnetic-field radiation relative to a benchmark configuration [52]. Casaucao et al. further showed that parasitic capacitance can modify circuit impedance, alter voltage and current, and shift the resonant frequency if it is not considered in the design process [56]. These results indicate that resonant conditions influence not only power-transfer efficiency, but also the electrical quantities that govern field radiation and parasitic-path excitation. High-Q resonant WPT systems are also sensitive to practical operating disturbances. Zhang et al. showed that the detuning level of the relay resonator affects system efficiency and directly determines rectifier operating mode [53]. Li et al. showed that, in a detuned multicoil structure, the transmitter-coil current depends strongly on the coupling condition, thereby changing the resulting magnetic-field distribution [55]. Casaucao et al. showed that parasitic capacitance modifies impedance and shifts the resonant point, thereby changing circuit voltage and current [56]. As summarized in Table 10, these findings indicate that off-nominal conditions can alter the electrical state of the resonant network and, consequently, its electromagnetic behavior.

Therefore, resonant design in high-frequency WPT systems should be evaluated not only from the standpoint of transfer efficiency, but also from the standpoint of EMC robustness under detuning, coupling variation, and parasitic-capacitance effects.

### 3.4. Parasitic Capacitances and Structural Coupling Mechanisms

In high-frequency WPT systems, the coupler structure contains not only the intended magnetic coupling path between the transmitting and receiving coils, but also unavoidable capacitive coupling paths. Parasitic capacitance is inherently associated with the physical configuration of the coupler and depends on factors such as coil geometry, coil size, spacing between coupled coils, and multilayer structural features [59].

**Figure 6:**
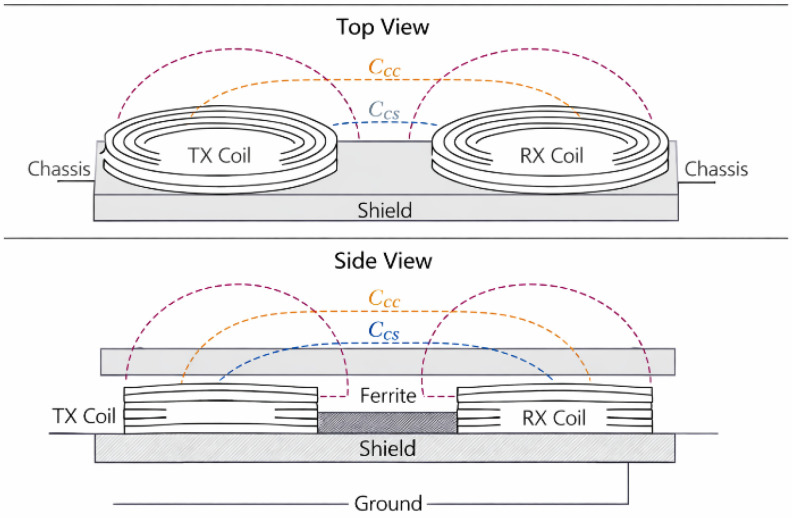
Conceptual parasitic-capacitance and structural-coupling paths in a high-frequency WPT coupler (vertical EV geometry).

From the above, Figure 6 depicts the standard vertical EV coupler arrangement, ground-mounted TX pad below, air gap, vehicle-mounted RX pad above, and illustrates the capacitive coupling paths (coil-to-coil, coil-to-shield, and shield-to-chassis) that arise within this structure at high frequencies. In practical IPT systems for electric vehicles, shielding metals are commonly placed below the ferrite layer to reduce electromagnetic field exposure, yet these elements also introduce additional parasitic capacitances to ground and affect common-mode behavior [60]. Mitigation strategies broadly fall into passive (ferrite and conductive plates), active (auxiliary current-driven coils), and reactive (resonant shielding coils) categories [61]. Kim et al. introduced the resonant reactive shielding concept for EV WPT systems, in which an LC-tuned auxiliary coil generates a counteracting magnetic field to reduce leakage in targeted regions [62]. Sun and Costinett later extended this approach to MHz-range operation by designing a resonant reactive shielding coil for a 3.03 MHz 500 W EV WPT system, demonstrating peak-flux-density reduction at the top of the coupler from 105 µT to 50 µT and side-leakage levels well below applicable safety limits [63]. Active shielding designs based on multicoil current injection have also been proposed for EV WPT systems; Cruciani et al. systematically optimized active shielding for automotive WPT, reducing leakage magnetic fields while preserving coupling efficiency [64]. Magnetic-shield design for SAE J2954-style Double-D couplers has been further investigated by Lei et al., who quantified shield loss and field distribution trade-offs in production-relevant geometries [65]. Beyond conventional shielding, metamaterial-based and metasurface-based field-shaping strategies operating at MHz frequencies, including hybrid-permeability metamaterial slabs, frequency-reconfigurable metasurfaces, and flexible metasurfaces, have been shown to simultaneously enhance coupling and reduce leakage outside the intended transfer region, providing an additional design axis for high-frequency WPT systems [66]. As a result, parasitic-capacitance effects in WPT systems should be interpreted not only at the circuit level, but also at the level of the coupler’s physical structure [60]. The main parasitic-capacitance paths in a WPT coupler are distributed across the physical structure rather than concentrated in a single lumped imperfection. A direct coil-to-coil capacitance exists between the facing TX and RX windings, and its magnitude depends on coil size, geometry, spacing between the coupled coils, and multilayer arrangement [59]. In EV IPT systems, the metal shielding plate located below the ferrite layer introduces coil-to-shield capacitances; once this shielding layer is grounded, it also creates a significant capacitive path between the transmitter coil and ground, which becomes part of the common-mode propagation network [60]. In addition, distributed capacitor placement and winding topology modify the local voltage distribution along the winding, thereby affecting the structure’s capacitive field behavior [67]. Accordingly, the parasitic-capacitance network of a WPT coupler should be treated as a structural coupling network rather than as a set of isolated parasitic elements, as Table 11 highlights.

Electrically, parasitic capacitances modify the effective impedance of the resonant network, alter the voltage and current, and shift the actual resonant frequency when they are not accounted for in the design [59]. In EV IPT systems, shielding metal placed below the ferrite layer introduces additional stray capacitances to ground and creates a low-impedance common-mode path [60]. Distributed capacitor placement also changes the local voltage distribution along the winding, thereby modifying electric-field behavior [67]. Therefore, parasitic capacitance in high-frequency WPT systems should be treated as an EMC-relevant electrical mechanism rather than as a minor circuit of non-ideality. These interactions introduce a clear design trade-off. Shielding can reduce field leakage, but the same grounded metal layer increases stray capacitance and can worsen common-mode noise behavior [60]. Likewise, distributed compensation can either mitigate or aggravate E-field emission depending on how capacitor placement aligns with the coil topology [67]. Coil optimization can reduce the effect of parasitic capacitance, but geometry choices still interact with the electrical behavior of the resonant structure [3]. Parasitic capacitances should therefore be regarded as part of the effective electromagnetic architecture of high-frequency WPT systems, and coupler geometry, shielding arrangement, and surrounding conductive structures should be treated as EMC design parameters.

## 4. Electromagnetic Interference Effects on Vehicle Systems and Surrounding Environment

**Figure 7:**
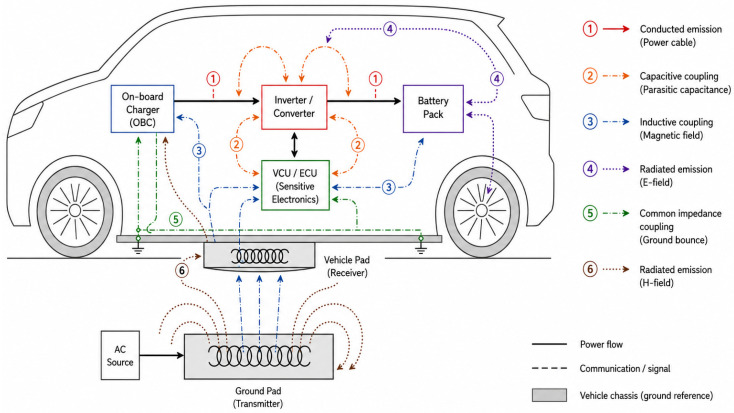
Conceptual illustration of electromagnetic interference coupling in an electric vehicle equipped with a wireless power transfer system.

High-frequency WPT systems operating in the MHz range add electromagnetic stress to the already dense electronic architecture of modern electric vehicles. Contemporary EV platforms integrate high-power inverters, battery management systems (BMS), advanced driver assistance systems (ADAS), and distributed communication networks, which increase susceptibility to electromagnetic interference in compact high-voltage environments [68]. Electromagnetic disturbances generated by fast switching transitions and time-varying fields may propagate through both radiated and conducted coupling paths within the vehicle structure. Communication buses, sensor modules, and electronic control units (ECUs) are therefore among the most EMI-sensitive subsystems in densely integrated EV architectures [68]. Quantitative investigations show that radiated EMI levels above 10 V/m are associated with increased bit error rates (BER) on CAN communication buses, that ECU resets were observed above 15 V/m, and that sensor outputs may deviate by up to 15% under elevated EMI exposure [69]. These results were obtained under controlled automotive EMI test conditions and demonstrate that EMI can directly affect communication reliability, control stability, and sensing accuracy in EV platforms [69]. Figure 7 shows the principal coupling paths between the WPT subsystem and surrounding vehicle electronics.

Radiated fields may interact with ADAS sensors, induced disturbances may propagate through communication wiring and ECUs, and eddy currents may be established in conductive body structures. Figure 7 therefore illustrates the source-path-victim relationship that underlies the functional and environmental effects discussed in the following subsections.

### 4.1. Impact on Electronic Control Units, Sensors, and ADAS Systems

WPT systems introduce additional electromagnetic stress into the electronic architecture of modern electric vehicles. Contemporary EV platforms integrate high-power inverters, battery management systems (BMS), electronic control units (ECUs), sensors, and communication networks, which increase susceptibility to electromagnetic interference in compact high-voltage environments [69].

High-frequency switching in modern power electronic converters generates steep voltage and current transitions, characterized by high dv/dt and di/dt, which are recognized as major drivers of conducted and radiated electromagnetic interference [70]. These switching processes produce both differential-mode (DM) and common-mode (CM) disturbances. In particular, common-mode interference is associated with rapidly changing voltage and parasitic capacitance to ground, whereas differential-mode interference is associated with pulsed current and stray inductance in the conduction path [70]. In inductive charging systems, magnetic fields are intentionally generated for wireless energy transfer. However, imperfect magnetic coupling and coil misalignment produce leakage fields outside the intended transfer region. These leakage fields do not contribute to useful power transfer and may instead act as unwanted electromagnetic interference sources. In addition, the WPT system geometry and test configuration influence interference paths and measured emission behavior, indicating that electromagnetic interaction depends strongly on installation conditions [71]. Experimental investigations have demonstrated measurable effects of radiated EMI on automotive communication and control systems. A significant increase in bit error rate (BER) on the CAN communication bus was observed when radiated EMI exceeded 10 V/m. ECU instability was additionally reported in the form of frequent resets when EMI levels exceeded approximately 15 V/m [69]. These results indicate that automotive control electronics can be vulnerable to elevated radiated electromagnetic exposure. Sensor subsystems are also affected under EMI stress. Experimental results show that accelerometer and temperature sensor outputs deviated by up to 15% from nominal values under elevated EMI exposure conditions. More broadly, sensors are identified among the electronic subsystems that may be affected by EMI in a dense EV electronic environment [69]. Susceptibility of the battery-monitoring chain has also been characterized: direct power-injection (DPI) and radiated tests on a representative automotive BMS IC reported by Crovetti and Fiori show that EMI on the cell-input and SPI lines can induce offsets in the acquired cell-voltage readings, and that the radiated field can drive the ADC into completely incorrect cell-voltage values across portions of the 1 MHz–1 GHz range [72]. ADAS sensing subsystems are particularly affected: automotive radar performance can be degraded by both intra-vehicle and inter-vehicle interference, with documented effects including loss of detection sensitivity, ghost-target formation, and reduced situational reliability [73]. Vehicle-level CAN communication is similarly EMI-limited; differential signaling provides intrinsic immunity, but EMI bursts can still corrupt several consecutive bits of a frame and trigger CRC and bit-monitoring errors that force retransmissions and reduce effective throughput [74]. Empirical studies of complete EV vehicles in different operating modes show that battery-charging conditions can produce broadband radiated emissions that exceed limits intended for residential, commercial, and light-industrial environments, highlighting that WPT systems must be assessed at the full-vehicle level rather than at the coupler alone [75]. Operational EMF surveys of high-power EV wireless charging installations confirm that electric and magnetic field levels around the body and inside the cabin depend strongly on vehicle geometry and coupler position, with measured electric-field strengths ranging from below 1 V/m to several tens of V/m and magnetic flux densities up to tens of micro tesla under typical charging conditions [76]. To summarize the affected subsystems and the corresponding interference effects, Table 12 presents a concise overview based on the reviewed findings.

From the source perspective, the principal EMI contributors in WPT-integrated EV architectures arise from fast-switching converter stages, leakage magnetic fields, and parasitic coupling with surrounding conductive structures. High dv/dt and di/dt transitions in the inverter stage generate CM and DM disturbances [70]. In parallel, imperfect coupling in the WPT coupler produces leakage fields, while coil offset and installation geometry modify field distribution and interference paths [71]. The electromagnetic behavior of WPT systems is therefore strongly influenced by operating conditions and system geometry. Variations in coil alignment, conductive surroundings, and supply-line arrangement alter interference paths, current distribution, and emitted field levels. This geometric dependence complicates repeatable EMC validation under practical vehicle-level conditions [71]. The main WPT-related EMI sources and their system-level implications are summarized in Table 13.

Together, Table 12 and Table 13 illustrate the source-path-victim structure governing EMI impact in WPT-equipped electric vehicles. The reviewed evidence shows that interference in such systems depends not only on converter switching behavior, but also on leakage fields, parasitic coupling, and geometric installation conditions. This supports the need for EMC assessment at the vehicle-system level [71].

### 4.2. Influence on the Electromagnetic Field Around the Vehicle

WPT systems generate alternating electric and magnetic fields in the vicinity of the TX and RX coils. Because the coupler has finite dimensions and a non-ideal spatial structure, part of the electromagnetic field extends beyond the intended transfer region, forming external leakage-field distributions around the charging pad and in the surrounding space [77]. Coil geometry is a key factor governing this behavior, since coil design directly affects field distribution, leakage characteristics, and overall electromagnetic performance [28]. Leakage-field magnitude and spatial localization are strongly configuration-dependent. In EV wireless charging systems, shielding, coil design, and field-shaping techniques are used to reduce electromagnetic leakage, but nearby conductive objects and surrounding structural conditions can still modify the resulting distribution, particularly for the electric-field component [28]. Misalignment between the transmitting and receiving pads further changes coupling conditions and redistributes the electromagnetic field, which alters the external leakage pattern around the charging structure. Because external field distribution is inherently non-uniform, exposure assessment cannot rely only on nominal operating parameters. Measurements at EV charging facilities show that recorded electric- and magnetic-field levels depend on charger configuration, measurement position, and operating condition, and local maxima must be identified experimentally rather than inferred from rated system values alone [70]. Numerical exposure modeling with realistic charger, vehicle, and human geometry likewise shows that field levels depend on spatial arrangement, distance, and surrounding structure [77].

**Figure 8:**
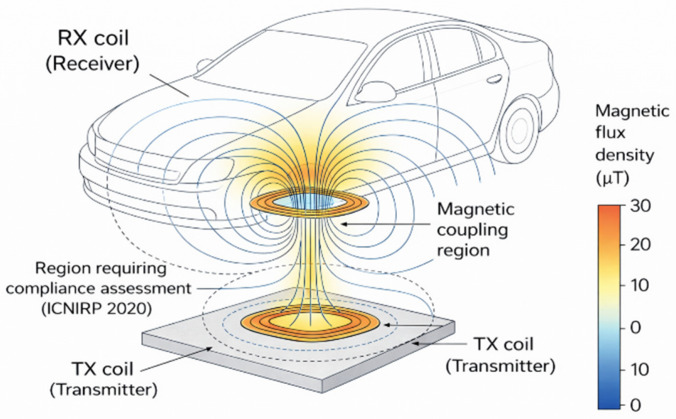
Magnetic field distribution around wireless charging coils of an electric vehicle in the MHz range.

Figure 8 illustrates the magnetic flux density distribution around the ground-mounted TX coil and the vehicle-mounted RX coil. Maximum field intensity occurs in the coupling region between the pads, while part of the field extends into the surrounding space as leakage flux. This external field region is the most relevant part to exposure assessment and electromagnetic compatibility analysis [78]. Table 14 provides a summary of the primary EMI sources in WPT systems.

The electromagnetic field distribution around EV charging systems is governed primarily by geometry, alignment, the surrounding conductive structure, and the observation position [28]. Therefore, field evaluation around the vehicle should rely on configuration-specific modeling and measurement rather than on simplified nominal conditions.

## 5. Discussion

### 5.1. Synthesis of Key Findings

The reviewed literature converges on three structural conclusions about MHz-range WPT for electric vehicles, summarized in Table 15. Across these findings, the recurring theme is that the transition from kHz to MHz operation is not a simple rescaling of frequency but a change in the electromagnetic regime, in which parasitic coupling and distributed-field effects come to dominate behavior that was previously controlled by magnetic coupling alone.

### 5.2. Core Trade-Offs

Three design trade-offs structurally constrain MHz-range EV WPT and recur across the reviewed studies (Table 16). Each pair demonstrates a clear benefit of higher-frequency operation with a corresponding electromagnetic penalty, so improving one objective typically requires a deliberate trade against another.

### 5.3. Open Problems and Future Research Directions

Despite significant progress in MHz-range wireless power transfer research, several important research gaps remain, limiting the transition from laboratory prototypes to large-scale electric vehicle deployment. One of the most evident gaps is the lack of standardized methodologies for measuring electromagnetic interference in MHz-range WPT systems. Many existing studies rely on custom experimental setups, different measurement bandwidths, or application-specific test conditions, making direct comparisons between published results difficult. Since most established EMC test procedures were originally developed for lower-frequency systems, their applicability to high-frequency resonant structures with strong parasitic coupling remains uncertain. This lack of harmonized evaluation procedures hinders objective benchmarking of mitigation strategies and complicates the development of universally accepted design guidelines.

One more important limitation is the relatively small number of experimental demonstrations conducted at full EV-relevant power levels. A large share of available studies focuses on reduced-scale prototypes or simplified laboratory environments that do not fully capture the complexity of real vehicle integration. In practical automotive platforms, conductive structures, wiring harnesses, metallic chassis components, and thermal constraints interact with the WPT system, strongly influencing electromagnetic behavior. Consequently, findings obtained under controlled laboratory conditions may not fully represent system-level EMC performance in realistic operating environments. More full-scale experimental validation is therefore required to bridge the gap between theoretical modeling and real-world implementation.

The combined influence of coil misalignment and high-frequency switching dynamics also remains insufficiently explored. While misalignment effects and inverter-induced EMI are frequently studied independently, relatively few works examine their interaction under dynamic operating conditions. In practical scenarios, geometric displacement alters magnetic coupling and field distribution, while switching harmonics to excite parasitic pathways. The interaction between these phenomena may lead to nonlinear emission behavior or unexpected resonance conditions that are not captured by isolated analyses. A more integrated investigation of geometric tolerance, converter operation, and electromagnetic emissions is therefore necessary to understand worst-case EMC scenarios.

A further research direction that has attracted rapidly growing attention concerns the use of magnetic metamaterials and metasurfaces in MHz-range EV WPT. Engineered structures with negative or near-zero effective permeability can act as magnetic field “lenses” that refocus leakage flux toward the receiver coil, simultaneously enhancing coupling, extending tolerable transfer distance, and reducing stray fields outside the intended transfer region [79,80]. Of particular relevance to the misalignment problem identified above, recent works have demonstrated that metamaterial slabs and spatially filtering magnetic metasurfaces can sustain transfer efficiency under lateral displacement and angular offsets that would otherwise sharply degrade conventional couplers, while frequency-reconfigurable metasurfaces enable adaptation of the resonant response to change geometric conditions [80,81]. From an EMC perspective, the same field-shaping mechanism can be exploited to confine leakage magnetic fields and reduce human exposure levels, providing an additional design axis that complements passive ferrite shielding and active cancelation coils. However, evidence at automotive power levels and MHz frequencies remains scarce: most reported prototypes operate at sub-kilowatt scale and ambient temperatures, and limited information is available on the structural integration of metamaterial layers into SAE J2954-compatible vehicle pads, their loss density under high-power excitation, and their interaction with surrounding ferrite and conductive shielding layers. Future research is therefore needed on low-loss and mechanically robust metamaterial layers compatible with production pad geometries, on the joint optimization of metasurface response together with resonant and active shielding strategies, and on tunable or adaptive metasurfaces capable of compensating misalignment in dynamic-charging scenarios without compromising EMC compliance. Together, these aspects place metamaterial-enabled coupler design among the most promising open avenues for reconciling power density, misalignment robustness, and electromagnetic compatibility in next-generation MHz EV WPT systems.

Human exposure and long-term electromagnetic field interaction inside the passenger cabin represent another area requiring deeper investigation. Existing exposure guidelines were primarily developed for lower-frequency systems and often considered simplified field distributions. In MHz-range WPT systems, the presence of electric-field components, complex vehicle geometries, and occupant proximity may lead to exposure patterns that differ substantially from conventional assumptions. Long-term exposure assessment, including variations in vehicle position, loading conditions, and shielding configurations, remains limited in the current literature, underscoring the need for multidisciplinary studies integrating electromagnetics, human safety modeling, and practical vehicle integration. Moreover, the interaction between high-frequency WPT systems and modern vehicle electronic architectures remains insufficiently characterized. Electric vehicles increasingly rely on advanced driver assistance systems, high-speed communication buses, and densely integrated sensor networks that may be sensitive to radiated or conducted disturbances. Although some studies report potential impacts on communication reliability and control electronics, systematic investigations of EMI coupling mechanisms between WPT systems and these subsystems are still scarce. As vehicle electronics continue to evolve toward higher levels of automation and connectivity, understanding and mitigating interactions among them will become increasingly critical. Taken together, these open problems define a clear research agenda for MHz-range EV WPT. Future work must move beyond component-level optimization toward system-level experimentation and cross-disciplinary analysis, with priority directions including: (i) the development of harmonized EMI measurement methodologies suited to MHz resonant structures; (ii) full-scale experimental validation at automotive power levels under realistic vehicle integration; (iii) integrated modeling of coil misalignment, switching dynamics, and parasitic propagation paths; (iv) updated human-exposure assessment and characterization of WPT interaction with on-board electronics; and (v) the maturation of metamaterial- and metasurface-enabled couplers as a co-design axis for misalignment tolerance, leakage-field containment, and EMC compliance. Progress along these directions will be essential for achieving reliable, regulation-compliant MHz-range wireless charging systems for electric vehicles.

## 6. Conclusions

This paper presented an EMC-focused state-of-the-art review of wireless power transfer systems for electric vehicles with particular emphasis on MHz-range operation. In contrast to conventional efficiency-oriented surveys and complementing earlier comprehensive EMC reviews of EV wireless charging, the present review adopted an EMC-first perspective and systematically examined electromagnetic interference sources, propagation mechanisms, shielding approaches, and their implications for regulatory compliance under realistic vehicle integration conditions. The analysis confirms that the transition to MHz operation is not merely a frequency increase but a fundamental shift in system behavior, in which electromagnetic compatibility becomes a primary design constraint alongside efficiency and power density.

The review identified several dominant EMI mechanisms that govern system-level EMC performance in MHz-range WPT systems. High-frequency switching transitions in GaN- and SiC-based inverters were shown to be major sources of conducted and radiated emissions due to high dv/dt and di/dt excitation of parasitic capacitances. In addition, resonant circulating currents, distributed electromagnetic effects, and coil misalignment contribute to leakage magnetic fields and broadened emission spectra. These mechanisms interact through conductive, capacitive, and inductive coupling paths, indicating that EMC performance cannot be understood by analyzing subsystems independently but must be addressed from an integrated system-level perspective.

A critical evaluation of shielding and field-control techniques demonstrated that both passive and active mitigation approaches can effectively reduce electromagnetic emissions, but each introduces trade-offs related to efficiency, thermal performance, system complexity, and physical integration constraints. Magnetic and conductive shielding reduce leakage fields but may increase losses and structural weight, while active or adaptive mitigation strategies improve flexibility at the cost of greater control and design complexity. These findings confirm that shielding optimization cannot be separated from converter design, resonant compensation, or mechanical packaging considerations.

The review also analyzed the inherent trade-offs between power density, system compactness, EMC, and human exposure compliance. Increasing operating frequency enables significant reductions in passive component size and supports compact system architecture; however, this same shift intensifies parasitic coupling and high-frequency emissions, often limiting practical performance gains. Consequently, the pursuit of higher power density increasingly conflicts with electromagnetic compliance requirements, highlighting the need for coordinated electromagnetic–thermal–control co-design approaches.

Several research gaps were identified that define future development priorities, consistent with the conclusions of multiple recent surveys of EV WPT. Existing studies are predominantly based on laboratory-scale prototypes and often lack standardized measurement procedures suitable for MHz-range operation, making direct comparison difficult. Experimental validation at full EV power levels remains limited, and the combined effects of coil misalignment, switching dynamics, and vehicle-level integration require further investigation. Additionally, current standards and exposure guidelines were largely developed for kHz-range systems and may not fully capture the electric-field components and complex coupling mechanisms characteristic of MHz operation. The limited availability of experimental data from full-scale MHz EV WPT systems further constrains the empirical basis in this domain, and further investigation is needed to consolidate the findings identified in this review. These limitations emphasize the need for harmonized test methodologies and more realistic vehicle-level assessments.

Based on the evidence reviewed, the main research question of this paper can be answered as follows: dominant EMI behavior in MHz-range WPT systems arises from the coupled interaction of fast-switching power electronics, resonant structures, and parasitic electromagnetic pathways, and effective mitigation requires system-level integration of shielding, compensation design, and converter operation rather than isolated optimization of individual components. The analysis further shows that EMC considerations fundamentally shape achievable power density and system compactness, making electromagnetic compatibility a key driver of architectural decisions in future high-frequency wireless EV charging systems.

The transition from kHz to MHz wireless charging represents a paradigm shift rather than a simple scaling of operating frequency. Future progress will depend on the development of holistic EMC-aware design methodologies that integrate power electronics, electromagnetic field control, shielding strategies, and regulatory constraints from the earliest stages of system design. Such an approach will be essential for achieving efficient, compact, and regulation-compliant wireless charging solutions that support next-generation electric mobility.

## Figures and Tables

**Table 1:** Detailed parameter comparison of conventional kHz-range and emerging MHz-range wireless power transfer for EV charging.

Characteristic	Conventional kHz-Range WPT	Emerging MHz-Range WPT
Operating frequency	20–150 kHz (SAE J2954 nominal 85 kHz)	MHz range, e.g., 6.78 MHz ISM band
Power-frequency scaling	Lower transferable power per coil area	Maximum transferable power scales with the square of angular frequency
Switching devices	Silicon IGBTs and MOSFETs	Wide-bandgap GaN and SiC devices enabling higher switching speed with lower loss
Passive components	Larger coils, capacitors, and heat sinks	Reduced passive size and higher power density
Field/circuit modeling	Quasi-static magnetic coupling; lumped-element model valid	Lumped-element assumptions weaken; parasitic capacitance and distributed effects are significant
Dominant EMI mechanisms	Mainly converter-centric conducted noise	Common-mode currents, high-Q resonant magnification, capacitive coupling, leakage-field redistribution
Stray parameters	Converter-centric, magnetic-coupling focus	Integrated coupler–converter–vehicle, frequency-aware EMC design

**Table 2:** Representative validity limits of classical IPT modeling assumptions at higher frequencies.

Classical Modeling Assumption	Main Limitation at MHz Frequencies
Magnetic near-field dominance	Electric-field and capacitive effects become more relevant
Lumped-element coil model	Distributed and parasitic effects become more important
Negligible radiation losses	Radiation-related effects may become more relevant
Negligible capacitive coupling	Capacitive coupling can affect system behavior and current paths; Negligible stray capacitance and stray inductance; Stray capacitance between coil turns, shielding, and chassis becomes significant; Stray inductance in interconnects produces distributed resonances and modifies common-mode current paths

**Table 3:** Comparative current behavior of compensation topologies in EV WPT systems.

Topology	Stray Parameter Sensitivity at MHz	Current Response to Reduced Coupling (M)	Main Operating Implication
SS	Highly sensitive: stray capacitance shifts resonant frequency; stray inductance in series path increases current stress	Primary-side current increases under reduced mutual inductance	High power capability but lower robustness under off-nominal conditions
LCL	Moderate sensitivity: shunt inductors partially absorb stray effects, but inter-winding capacitance can alter impedance matching	More stable current behavior	Improved robustness but lower transferred power
LCC	Moderate-to-high sensitivity: additional reactive components increase the number of stray coupling paths	Robust behavior with higher transferred power than LCL	Good robustness with improved transmission performance

**Table 4:** Representative functional stages of an EV WPT system and their EMC relevance.

Subsystem	Impact on EMI/EMC
AC/DC Converter	Defines front-end power conditioning and harmonic behavior
DC link	Couples the front-end stage to the high-frequency power stage and participates in conducted noise propagation
High-frequency inverter	Main active switching-noise source due to large dv/dt and di/dt
Resonant compensation network	Determines resonant voltage and current distribution

**Table 5:** Dominant EMI propagation paths in MHz-range WPT systems.

EMI Propagation Path	Description
Conductive paths	Disturbance propagation through wired interconnections and CM/DM return paths
Capacitive paths	Electric-field coupling through parasitic capacitances associated with coils, shielding structures, and nearby conductive parts.
Inductive and field-leakage paths	Magnetic-field coupling and leakage-field interaction originating from the TX/RX coupler.

**Table 6:** Selected material properties of silicon (Si), GaN, and SiC are relevant to power-device performance.

Parameter	Si	GaN	SiC
Bandgap $E_{g}$ (eV)	1.12	3.39	3.26
Critical electric field $E_{\mathrm{crit}}$ (MV/cm)	0.23	3.3	2.2
Electron mobility $\mu_{n}$ (cm^2^/V·s)	1400	1500	950

**Table 7:** Influence of switching parameters on high-frequency noise excitation.

Switching Parameter	Effect on Spectral Magnitude	Effect on High-Frequency Behavior
Increase in DC-link voltage	Increase in overall noise-source spectral magnitude	No major shift in spectral corner frequencies
Increase in switching frequency	Redistribution of spectral content with higher repetition of switching harmonics	Upward shift in the lower spectral corner
Reduction in rise time	Increase in high-frequency spectral amplitude	Upward shift in the upper spectral corner toward the MHz range
Increase in dv/dt	Increase in common-mode and differential-mode high-frequency noise amplitude	Extension of high-frequency spectral content and stronger excitation in the MHz range

**Table 8:** Reported influence of misalignment on magnetic coupling.

Misalignment Type	Observed Effect	Quantitative Result
Rotational misalignment	Reduction in coupling coefficient	Minimum k = 0.266
Rotational misalignment	Mutual inductance fluctuation	2.3% variation
Axial displacement	Mutual inductance variation	6.3% fluctuation
Practical misalignment tolerance	Efficiency degradation observed	Measurable coupling reduction

**Table 9:** Resonant current magnification in high-frequency WPT systems [56,57,59].

Observation	Physical Implication
Coil current is treated as a system-level design variable linked to magnetic flux density	Internal resonant current directly influences field generation
Relay-resonator detuning changes efficiency and rectifier operating mode	Resonant current level depends on tuning state
Maximum secondary current occurs under resonant compensation conditions	Tuned operation can increase internal current stress

**Table 10:** Resonant conditions relevant to EMC behavior in WPT systems.

Condition	Effect on Resonant Behavior	EMC Relevance
Relay-resonator detuning	Changes efficiency and rectifier operating mode	Operating-state changes can redistribute current and voltage stress
Coupling-dependent current redistribution in detuned multicoil systems	Changes transmitter-coil current and the magnetic-field distribution	Weakly coupled coils can still contribute to stray field unless the current is attenuated
Parasitic capacitance	Modifies impedance and shifts the resonant frequency	Resonant response becomes more sensitive to parasitic current paths

**Table 11:** Main parasitic-capacitance paths in the WPT structure.

Path	Physical Origin	Main Structural Role
Coil-to-coil capacitance	Facing TX and RX windings	Direct capacitive coupling between the power coils
Coil-to-shield capacitance	Winding layer above the shielding plate	Couples the coil to the shielding structure
Coil-to-metal plate/ground capacitance	Grounded shielding metal below the ferrite layer	Forms a low-impedance common-mode path to ground
Capacitance to grounded conductive structures	Ground-referenced conductive parts associated with the IPT assembly	Extends the structural capacitive propagation path beyond the windings
Distributed capacitance in segmented or multilayer layouts	Segmented compensation, multilayer windings, and topology-dependent winding arrangement	Alters local voltage distribution and capacitive field behavior

**Table 12:** Vehicle electronic subsystems and reported EMI effects.

EMI Source	Physical Mechanism	System-Level Consequence
SiC/GaN inverter switching	High dv/dt and di/dt transitions	Generation of CM/DM conducted disturbances
WPT coil magnetic leakage	Imperfect magnetic coupling	Radiated interaction with vehicle electronics
Parasitic coil–chassis capacitance	Capacitive CM coupling	Common-mode current propagation to chassis
Coil misalignment	Geometry-dependent field redistribution	Variation in emission levels

**Table 13:** Representative EMI-sensitive subsystems in WPT-equipped EVs and their associated coupling mechanisms and effects.

Subsystem	Dominant Coupling Mechanism	Reported Effect
ECU	Radiated	Reset observed when EMI > 15 V/m
CAN bus	Radiated	Increased BER when EMI > 10 V/m
Accelerometer/Temperature sensor	Radiated	Output deviation up to 15%
Sensor electronics	Radiated	Susceptibility to EMI-induced disturbance
Wiring/return-path structures	Common-mode propagation	EMI transfer through CM coupling paths

**Table 14:** Factors influencing magnetic field distribution in WPT systems and their implications for EMC assessment.

Parameter	Effect on Field Distribution	Assessment Implication
Coil geometry	Determines flux confinement	Influences the leakage magnitude
TX–RX alignment	Alters spatial flux redistribution	May shift local maxima
Surrounding conductive structures	Modify field paths	Can increase localized peaks
Charger configuration	Affects measured field levels	Compliance depends on the measurement position
Vehicle–charger geometry	Governs near-field distribution	Requires simulation-based validation

**Table 15:** Key findings synthesized from the reviewed MHz-range EV WPT literature.

Domain	Key Finding	System-Level Implication
Electromagnetic regime	Parasitic capacitances, common-mode paths, and distributed-field effects dominate over pure magnetic coupling at MHz frequencies.	EMI arises from coupled interaction of inverter, coils, compensation network, vehicle grounding, and surrounding conductive structures, not from any single subsystem.
Wide-bandgap devices	GaN/SiC fast switching enables MHz operation, but steep dv/dt and di/dt intensify common-mode excitation and broaden the emission spectrum.	The same device characteristics that support high power density also make EMC control more demanding.
Integrated coupler design	Coil geometry, compensation topology, shielding, and nearby conductive structures must be treated as one electromagnetic system.	EMC performance cannot be reached through isolated subsystem optimization; it requires integrated system-level design.

**Table 16:** Core design trade-offs in MHz-range EV WPT systems.

Trade-Off	Benefit	Cost	Design Implication
**Power density vs. EMC**	Compact coils and reduced passive volume at MHz operation.	Stronger parasitic coupling, increased sensitivity to layout and vehicle structure, and broader high-frequency emissions.	Frequency scaling improves compactness but can erode the net advantage of higher-frequency operation unless EMC is co-designed.
**Field containment vs. efficiency and mass**	Improved compliance with leakage-field and human-exposure limits via shielding or field-shaping.	Additional losses added to the shielding mass, thermal penalties, and more complex control.	Better field containment is rarely free; the cost reappears in efficiency, packaging, or design complexity.
**Switching speed vs. emission** **control**	High switching frequency, fast transitions, and reduced converter volume enabled by GaN/SiC devices.	Higher conducted and radiated disturbances and stronger common-mode excitation from steep dv/dt and di/dt.	Converter speed and electromagnetic cleanliness are coupled; both must be addressed at the design stage by explicit parasitic-path management.

## Data Availability

No new data were created or analyzed in this study.
